# Religious Leaders in Politics: Rio de Janeiro Under the Mayor-Bishop in the Times of the Pandemic

**DOI:** 10.1007/s41603-020-00123-1

**Published:** 2020-10-09

**Authors:** Renata Siuda-Ambroziak, Joana Bahia

**Affiliations:** 1 Institute of the Americas and Europe, American Studies Center, University of Warsaw (UW), Warsaw, Poland; 2 State University of Rio de Janeiro (UERJ), Rio de Janeiro, RJ Brazil

**Keywords:** Religion, Politics, Leadership, Rio de Janeiro, COVID-19, Pandemic, Religião, Política, Liderança, Rio de Janeiro, COVID-19, Pandemia

## Abstract

The authors discuss the phenomenon of religious and political leadership focusing on Bishop Marcelo Crivella from the Universal Church of the Kingdom of God (IURD), the current mayor of the city of Rio de Janeiro, in the context of the political involvement of his religious institution and the beginnings of the COVID-19 pandemic. In the article, selected concepts of leadership are applied in the background of the theories of a religious field, existential security, and the market theory of religion, to analyze the case of the IURD leadership political involvement and bishop/mayor Crivella’s city management before and during the pandemic, in order to show how his office constitutes an important part of the strategies of growth implemented by the IURD charismatic leader Edir Macedo, the founder and the head of this religious institution and, at the same time, Crivella’s uncle. The authors prove how Crivella’s policies mingle the religious and the political according to Macedo’s political alliances at the federal level, in order to strengthen the church and assure its political influence.

## Introduction

The COVID-19 has become the main global issue of 2020. Reducing its effects on people, social relations, and economic sector not only has turned into a major challenge for any political leader but also has reminded of the existing limitations in terms of human capacity and organizational structures, making strong and weak points in leadership styles and crisis management much more visible. At the same time, the epidemic has been also used for exacerbating political games in many countries, including Brazil, with politicians building new electorate bases, changing allies, or actively defending their position. In such difficult circumstances, the demand for leadership addressed simultaneously to minds and emotions has become clear—belief in the powers, charisma, and sometimes almost magical forces of many leaders has become more than perceivable, and the emotions have been overflowing from religious sphere onto the political one and vice versa.

Religiosity, sensibility, imagination, and the faith in miracles sometimes have such intensity and force that they contribute to interference of religious sentiment in political judgment and opinion. In Brazil, the affective appears in the very concept of authority, founded on the faith in the superiority of charismatic leaders (both religious and political), supposed to provide safe haven in times of difficulty, especially for the most numerous social groups, whose status is of the excluded and depreciated. In this sense, the existence of religious leaders in the political sphere emanates from, but also emphasizes, various problems, including social, political, and economic tensions, which constitute an important factor in choosing the option these leaders embody among many available alternatives (religious and political) and supporting their alliances and visions.

There exist multiple perspectives from which the complex phenomenon of “leadership” can be analyzed both in political and religious contexts (Rost 1991; Stogdill 1974; Burns 1978; Ciulla 2004; Edwards 2000; Goleman 1998, 2000; Siuda-Ambroziak 2017). Rost (1991) analyzes 221 concepts of leadership, and Stogdill (1974: 259) states that “there are almost as many definitions of leadership as there are people who try to define the content of the concept.” The fact that leadership is often combined or closely identified with religion, politics, power, and influence makes it even more difficult to define, although there are some universal threads that form the basis of its understanding.^1^ The perception of leaders is also always through the prism of the time in which they act, the qualities they possess, and skills and abilities that allow them to gain support and consolidate their relationship with the followers (Goleman 2000; Myers 2007).

A leader, for the sake of the paper, is defined by us as a person who influences others through one’s actions and decisions and possesses institutionalized possibility of enforcing procedures, legal regulations, etc. due to performing specific functions and occupying a specific position in the social and political structure, especially by means of elections and democratic mandate (the position of the mayor). We acknowledge the fact that a leader is strongly supported in electoral campaign by his interest group (for instance a religious institution, here, IURD), which later may control and pressure him during his office. Such leaders are institutionally empowered (by their political party or/and their church)—they are put forward to bring their group closer to achieving the desired objectives (expansion and political influence). What helps such leaders in performing their tasks are recognition and support offered by more influential members of the same group (the relationship between bishop Macedo, the founder on the IURD, and bishop-mayor Crivella, his nephew).

Leadership can appear at different levels of our social life: microstructural level, i.e., in interpersonal relations; mesostructural level, i.e., mainly in middle-sized social groups; and macrostructural level, entangled in a deeper context of socio-economic and political relations and the play of interests of large social groups. And this is precisely the level that is most interesting to us in this paper, which exposes some of various dimensions of power, its sources, conflicts stemming from exercising it, and their results. This concept of leadership in the wider public sphere (and in politics above all), in the turbulent periods of crisis (like the current pandemic), might require the use of some extraordinary means (Burns 1978)—the majority of the followers in such circumstances are weak and insecure and they want their leader to represent their group by articulating vision, giving reinforcement, and showing self-confidence, determination, and courage in its implementation. Effective leadership is in such cases essential as it provides the supporters with a sense of security. Greenleaf (1977), presenting the concept of the “caring leadership,” argues that the primary duty of a leader should be to help, care for, and meet the needs of the followers, especially in difficult times of various macro-problems (here, pandemic, corruption in political life, economic crisis), which increase the leader’s popularity and authority, if combined with his self-confidence in taking and implementing the expected by followers decisions.

Our analysis ensues discussing various intersections between religion and politics in terms of leadership and policies implemented in the city of Rio de Janeiro by the IURD bishop-mayor, Marcelo Crivella, provoked especially by unexpected discontinuities and ruptures due to the COVID-19 pandemic, analyzed in the context of the church strategies of growth by means of political alliance implemented by its founder and Crivella’s uncle, Edir Macedo. Using as theoretical references, Bourdieu’s (1991) ideas of correspondence between religious options and social groups and the existence of the “religious field,” we look at the political decisions of Marcelo Crivella—an important religious leader currently in public office to see the meaning and the results of the city hall–promoted policies for various social, economic, and political groups in the context of their competition or cooperation.

In our analysis, we apply also the ideas of Stark and Bainbridge (2007), who base their market theory of religion on the economic principle of a rational consumer choice, maximizing profits, and minimizing losses in following a religious leader or adhering to a religious institution, perceiving human behavior in the realm of religion in terms of beneficial exchange: religious leaders (or institutions) offering their followers/members rewards (tangible and practical benefits) and compensators, i.e., religious doctrines which promise relief in facing adversity and salvation, and followers—incurring the costs (paying a real tithe) of their participation in the entire socio-cultural system created in this way, also linked to politics. The benefits which are given or expected by individuals foster their conversion and, subsequently, electoral decisions in which the strategic role is played by religious leaders, who can prove their powers, efficiency, and negotiation skills not only in a purely religious context.

Religion, economics, and politics have always been inextricably intertwined. Especially during times of serious social, economic, and political crisis, as the one Brazil has been facing due to the current pandemic, a great potential stems precisely from leaders (including the religious ones), acting as an organizing and mobilizing force for the masses. In difficult times, people often turn to their faith communities, looking up to their leaders for help, hope, and also being practical not only eschatological solutions and advice. According to Norris and Inglehart (2006) and their compensational theory of existential security, the strength of religious leaders and their organizations depends mostly on the perceivable security level of its members and the dominant type of religious culture, including the social perception of religious leaders and their roles. Religion is viewed as a factor of the psychological mitigation of risk, assuring people that even though they are not able to understand or predict what is to come, *force majeure* will watch over them, which lowers stress level and reinforces their sense of security. This means that an augmented need for religion appears always when people experience severe deprivation, stress, or a direct threat to life or health, and religious solutions appear because of the lack of possibilities to eliminate their source, which is exactly the case of the COVID-19 pandemic, spread around the globe and through Brazilian nation, making it the second most targeted in the world in numbers,^2^ and Rio de Janeiro—the second city hardest hit by the pandemic in Brazil.^3^ In the situation of insecurity and strong mental tensions, sometimes the only option is to entrust one’s fate to Providence or—its earthly representatives. This explains why the degree of engagement in religious practices in poor, unequal, war-torn, or natural disaster–vulnerable societies is always higher than in the richer, more equal, and safe ones—the higher gross domestic product (GDP) per capita of a country^4^ and the lower the Gini coefficient,^5^ the lower the religious involvement, with the weakest social groups being the most religious element in the structure of any society. Nowadays, they are precisely the ones who, in the pandemic situation, have no means to protect themselves and their families. In such cases, religious legitimization comes to the fore, especially when combined with the impact of active proselytizing, such as the one adopted by the IURD, with its pragmatic policy of growth and strategies of increasing influence in the secular world, implemented via the investment in the media and in politics (Mariano 1999; Oro 2005-2006; Mariano and Oliveira 2009; Siuda-Ambroziak and Stachowska 2017).

In the view of the above, we focus in our paper on bishop/mayor Crivella’s political career in the context of his church political involvement and his behavior in response to the crisis, and we analyze how he, as a religious and political leader, deals with various challenges of the city management, including the ones provoked by the current pandemic. In our analysis we look, first of all, at accessible current information, including online news and institutional websites, social networks, etc., and comment on the interface between the world of religion and politics, especially in the context of the COVID-19-struck Rio de Janeiro. In order to complete the picture, we mention the IURD historical strategies of growth in the political field, analyze circumstances that shaped Marcelo Crivella, allowing him to rise to the office of the mayor, and show how he is exercising his democratic mandate as the bishop/mayor, including processes, phenomena, and events dependent (resulting from his decisions) or independent of him/her (resulting from unpredictable factors, like the pandemic). When presenting and evaluating the bishop/mayor’s decisions in the pandemic circumstances, we take into account a number of criteria: choices of action and its implementation, political support and alliances, and mobilization of target groups.

## The IURD in Brazilian Politics—Strategies of Growth

The IURD was registered in 1977 as Igreja Universal do Reino de Deus by Edir Macedo (initially in cooperation with his brother-in-law). The church started gaining visibility during the difficult democratic transition after 1985, marked by the implementation of tough neoliberal reforms. Neo-Pentecostals, due to their cult of pragmatic business management techniques and the spirit of entrepreneurship, soon became “the third horseman of neoliberalism” for having adapted so well to economic and political transformations and taking advantage of them to the benefit of their own expansion. As a result, the IURD, an innovative “religious enterprise” according to Stark and Bainbridge theory, the Macedo family owned and managed, eclectic in doctrine, cult, and organizational structure, started also increasing its political influence. The strategic role in the institutional expansion has been played by its charismatic founder and leader and his closest collaborators, including Marcelo Crivella, who early joined his uncle in this endeavor. They worked out advertising strategies, proposed attractive and flexible doctrines formed by means of experimental syncretization (Mariano 1999; Oro 2005-2006), developed techniques of church management, and started their expansion in the area between economy, politics, and religion or, in other words, money, power, and faith (Siuda-Ambroziak 2019). IURD, according to Stark and Bainbridge’s theory, sells religious compensators, which are a syncretic set of the most attractive ingredients in a particular market. This is the easiest and most effective way to generate “religious bestsellers” by skillfully combining what is effective in the system in new configurations and adding to it entertainment, community, or political involvement in order to be able to control as many aspects of the members’ life as possible, including leisure and public activities. An interesting aspect of the IURD’s strategy is also the church’s attitude towards money donated by the followers as a way of fulfilling the traditional “reciprocity rule” - it becomes to the church a proof of the effectiveness of its own teaching, providing fuel for its further expansion. In the church discourse, there is no possibility to be saved “for free”—the “ethics of prosperity” promotes a vision of wealth as a visible sign of God’s blessing (which is shown in the lavish lifestyle of the bishops in luxurious “havens for millionaires”)^6^ and encourages its followers to develop the entrepreneurship spirit and enrich (Siuda-Ambroziak 2019), thus raising their social status and community prestige (a phenomenon known as “empowerment”).

Stark and Bainbridge (2007) observe that religious innovations often achieve a “high productivity rate,” successfully adapting to their market conditions and appearing at the precise timing. The IURD appeared with its innovative proposal at the perfect historical moment of transition and crisis and skillfully used them to its great advantage. As a good example of an “innovative cult,” IURD has been effectively organized and managed, with its activity based on attractive religious product delivery, collection of payment (in case of IURD it is a real tithe and additional donations), and generation of profit, reinvested in further organizational expansion. Macedo must have known from the beginning the benefits coming from activity in the religious field—before he set up the IURD, he had been a member of the first in Brazil Neo-Pentecostal church (*Nova Vida*), where he did not manage to start a pastor career due to his personal conflict with the church’s founder—McAlister.^7^ It is also worth reminding that before the foundation of IURD, Macedo had already tried to establish another church—*Cruzada do Caminho Eterno*, which did not prove to be successful, though. IURD was his third attempt to set up a successful hybrid church, and it became finally a profitable investment, quickly transformed into an efficient and expansive structure (Ferrari 2007). Assuming that his offer would be successful only when he finds a sufficiently numerous group interested in its uphold, Macedo focused on poor urban *favela* inhabitants. In fact, basing on the assumptions of Norris’ and Inglehart’s theory, which explains the success of a given religion in the society by the low level of existential security, we can infer that high levels of poverty, marginalization, crime, social disparities, economic and political turmoil, and, nowadays, pandemic have been all providing extra demand for the IURD religious proposal and more fuel for its further expansion, requiring charismatic leadership of quasi-shamanic nature, able to deal with demons and offer instant healing.

Macedo set up a vertical hierarchy in his church, just like the one he knew from the Catholic church (which was his first affiliation), becoming the infallible “pope” and introducing strict rules of obedience to his authority. IURD, like other innovative enterprises, has been set pragmatic goals—it makes good investments to make profit, which has already resulted in building a genuine international business holding, involving not only religious field but also the media market, entertainment sector with a record studio, and financial services sector (Siuda-Ambroziak 2019). The “faith empire” is a family business, co-run by his wife, two daughters, and two sons-in-law (both pastors), of which one is indicated as a possible successor. Their blogs are put on the websites of the radio stations side-by-side with Macedo’s. This prepares ground for the closest family members to take over the empire in the future (Castro 2017). However, the succession is being prepared also in terms of preserving and strengthening the IURD influences in the national, political field, and here Marcelo Crivella’s candidacy seems to be the strongest.

IURD is a very pragmatic institution—presenting the testimonies of those who have succeeded, the church raises hope of prospective converts, but at the same time does not give any guarantee of success, knowing the marginalization and exclusion mechanisms underlying the structure of the Brazilian society. Instead, it allows the followers to identify vicariously with the successful expansion of their church—that is why huge religious gatherings, not only of proselytizing nature but also aiming at strengthening the support of the political allies during campaigns, are organized by IURD with an intention of exchanging political and religious capital (Dilma Rousseff’s presence at the official inauguration of the Temple of Salomon, in 2014, before her reelection; president Bolsonaro’s presence at the March of Jesus, in 2019, in São Paulo; presence of mayor Crivella and president Bolsonaro at the 40th anniversary of the International Church of the Grace of God in February 2020, in the city of Rio de Janeiro); construction of monumental temples (like the Catedral del Castilho in Rio de Janeiro and Temple of Salomon in São Paulo); institutional representatives in the positions of political power (since 2003, there has existed a political party linked directly to IURD, the Brazilian Republican Party)^8^; and a bold missionary zeal with Macedo’s vision of creating the universal structure, covering all continents.^9^

The most interesting to us in this paper are the strategies of the IURD expansion in the public sphere by means of the investments in national politics started with the presidential elections won in 1989 by Fernando Collor de Mello, officially supported by bishop Macedo. The IURD, since that time, has been working on its own leaders’ and followers’ political careers, especially after purchasing a Record TV station, which soon became the second largest television channel in Brazil. Its proselytizing and political impact grew so much that bishop Macedo allowed himself to publicly criticize presidential decisions and to withdraw his support from the electoral campaign. In such circumstances, in 1992, he was arrested under the accusation of charlatanism and tax evasion. Due to the lack of evidence and numerous protests and demonstrations in his defense, he was released after a few days, but his ordeal only helped his popularity. Soon president Collor, involved in a corruption affair, was unseated by the first in Brazil’s history impeachment procedure.

Already during the next presidency, of Fernando Henrique Cardoso, Rio de Janeiro became the most (Neo)Pentecostal among all Brazilian cities—between 1992 and 1994, 21% of all its inhabitants were converted (Fernandes 1998). IURD popularity was growing systematically with religious meetings held at all major football stadiums in Brazil. At the end of the 90s, bishop Macedo started construction of the monumental Cathedral Del Castilho in Rio and realized new investments in the media sector by purchasing radio broadcasting stations, grouping them all under the religious holding “Rede Alleluia-Rede da Família,” and setting up his own publishing house, systematically developed (Siuda-Ambroziak and Stachowska 2017). In the presidential elections of 2002, Macedo officially supported Luiz Inácio Lula da Silva (Lula), the leader of the *Partido dos Trabalhadores*—*PT*, in spite of having accused him earlier of postulating dangerous populism (Alves 1998; Santos and Da Escóssia 2002). In the meantime, he started to introduce a strategy of increasing the number of his church representatives at various political levels using his media for the sake of their campaigns—in 2002, Macedo bought a radio station in São Paulo (99,3 FM), equipped with the highest standard studios broadcasting via satellite. Earlier already, he had made one of his bishops, Carlos Alberto Rodrigues Pinto, responsible for political alliances and, subsequently, successfully supported Marcelo Crivella in federal elections for the Senate.

Macedo supported Lula in winning his second term, and the cooperation between the two leaders was visible on all fronts, for example, Macedo, his family, and closest collaborators received diplomatic passports and Lula supported Macedo in his warfare against the media competitors, eagerly learning from him as well about the successful fundraising techniques for the sake of his political party (Weis 2008). Bishop Macedo helped also in the presidential campaign of Lula’s successor, Dilma Rousseff, who owed him a lot due to the loss of the Catholic church support caused by her apparently “pro-abortion views” (Siuda-Ambroziak 2019; Siuda-Ambroziak and Stachowska 2018) and he appeared among honorary guests at Dilma Rousseff’s presidential inauguration. At that point it became clear that his followers constituted already a solid political force, difficult to ignore, using their votes according to their religious leaders’ indications. It was also visible at the moment of Dilma Rousseff’s final fall, this time unassisted by Macedo, and her impeachment on the charges of budget manipulation in the context of deep economic recession and social frustration with the levels of corruption, which culminated in massive and unprecedented street protests. The church has already had two important political representatives before the beginning of Crivella’s mayor term: that of the minister of fishing and aquaculture, represented by Marcelo Crivella himself in Dilma Rousseff’s government, and that of industry, foreign trade, and services with another IURD bishop—Marcos Pereira (in Michel Temer’s government), who is possibly the future head of the Congress, currently its 1st vice president and the president of the Republican Party.

In 2018 presidential elections, Macedo officially backed up the candidacy of Jair Bolsonaro against the successor and the nominee of at that time imprisoned Lula and impeached Dilma and continues supporting the federal government up till now, also by means of the political decisions of the IURD representatives in local governments, including Crivella in the Rio de Janeiro city hall.

To sum up, we might notice that IURD development and expansion have shown strong interdependence between the country’s economic, political, and social situation and the demand for its offer, especially among the most vulnerable groups, including the current pandemic exposure, social inequality, discrimination, and violence. We have also shown that the church strategies of growth have included careful preparation and flexible adaptation of the religious offer to the needs of the followers; creation of centralized, well-managed institutional structure; promoting strong, charismatic leadership, together with investments in various sectors of the market with the stress on the media; entering the world of national politics at various levels by means of putting forward own candidates in elections, providing them with full institutional support; and forming important political alliances at all governmental levels.

## The Man and His Circumstances—Marcelo Crivella’s Pre-Pandemic Background

Marcelo Crivella, a bishop of the IURD, and its founder’s nephew, was elected mayor of the city of Rio de Janeiro in 2016, beginning his mandate in January 2017. His political career has been from the beginning closely related to the TVRecord/IURD/Republican Party conglomerate, a media-religious-political structure created by the IURD and currently allied with the government of Jair Bolsonaro.

In his youth, Crivella was affiliated with the Methodist Church, but due to his close relationship with his uncle, Edir Macedo, he soon changed it for the sake of the IURD. After conversion, he started working for the church and, having his ministry approved, he became a pastor and then was consecrated to bishop. He worked for 10 years as a missionary in African countries. His religious preaching has been very often broadcast nationally, and he became a famous religious singer and composer, having 14 records released on Line Records (belonging to Record) with over 5 million copies sold.^10^

Crivella’s entry into politics is a result of a religious political movement sometimes called “Pentecostal eruption” present in politics since 1986 (Freston 2006), in Brazil noted with the participation of the Evangelicals in the election of Collor in 1989 (Mariano and Pierucci 1992). Crivella was elected senator for the first time only in 2002, with about 3.2 million votes, having as his political “godfather” Garotinho,^11^ who persuaded him to curb his religious tone, which was necessary for advancing in the political activity. His political campaign was based on the success of his charity project in the semiarid zone of the Northeastern state of Bahia, where, according to the official IURD site, he settled down ca. 100 families, irrigated the area, and sustained it with the money coming from the sale of his records.^12^ Afterwards, he tried, in vain, with the presidential support of Lula da Silva, to become the mayor of Rio de Janeiro in 2004, the governor of the state of Rio de Janeiro in 2006, for the second time the mayor in 2008, and, finally, in 2010, Crivella became the first senator reelected in the state of Rio de Janeiro in 24 years. He held office until he was sworn in as mayor of the capital of the state of Rio de Janeiro, elected in 2016 in the second round, in dispute with the leftist Marcelo Freixo, of the PSOL political party.^13^

Some analysts attribute his victory to a good time setting, in which conservative forces were growing in the country, added to a vacuum on the left side of the political scene, errors of strategy in the case of other candidates, and also a weak political agenda of his opponent in the second round.^14^ In addition to using secular discourse at times when it was necessary to win another portion of the electorate, Crivella sometimes had to distance himself during his campaign from the IURD, of which he continues being a licensed bishop, and adopt the “governing for all” strategy. He also limited his speeches on the issue of religious intolerance, affirming in his campaign that the fact of being Pentecostal did not imply that he would persecute other religions, including of African origins. To this end, Crivella used a discourse that his church was a religious minority itself, so he would not persecute other minorities, and also the idea that Brazil is a secular state (though not atheist). Camurça (2020) shows that the idea of religious minority is the key to understanding the social and religious identity of the IURD and its activity in public space—the uses of the term are strategic, sometimes demonstrating autonomy, liberalism, and modernity of the church and sometimes opposing Catholics as an oppressive religious majority in Brazil.

Crivella’s mandate is a result of a long-lasting Pentecostal and neo-Pentecostal religious segments’ investment in politics, as well as of an increasing dialectic relations between politics and religion in Brazil (Mariano and Oliveira 2009), with the strategy of supporting allies and running for political positions with a conservative agenda bearing ripe fruits in Crivella’s campaign organized with the motto “I will take care of you,” evoking an idea of a specific religious social welfare, in which the population is under the protective wings of their pastor-shepherd. Such an idea was especially warmly welcome in poverty-and violence-stricken communities where Evangelical denominations are rapidly expanding in the context of the State notorious absence. The “social issue” has been, in fact, the usual core electoral agenda of Crivella, not only during the campaign for the city hall of Rio de Janeiro—the implementation of various social assistance projects and social rhetoric is one of the main strategies used by the IURD in order to win social support, ease possible controversies, and promote an idea of social dialog in the process of conquering public space beyond the religious sphere. The motto of Crivella was also a well-prepared way of criticizing, between the lines, the city management of the previous mayor, Eduardo Paes, who prioritized works rather than people, so the idea of human to human care became a good political slogan for the campaign.^15^ At the same time, he tried also to subtly soften his image as a IURD bishop to attract the voters of other churches and religions. Despite having a strong support from all Evangelical segments, Crivella, according to Machado (2006), did not want to restrict himself to the (neo)Pentecostal public, and he did eventually manage to expand his electorate beyond his religious brethren. This long-term, successful strategy had already made him gain political space throughout several elections before the 2016 campaign for the mayor of Rio de Janeiro—he has always adopted, on the one hand, political pragmatism but, on the other hand, a moderate approach in many controversial matters, which is a typical policy applied by the IURD when promoting its political candidates, project, and investments^16^ (Camurça 2020; Burity 2018; Mariano 1999). At one point, Crivella apologized for his homophobic statements in the past, pledging to maintain the city’s funding for the Gay Parade and the Carnival, confirming his deep respect for all kinds of minorities’ demonstrations and promising to finish off with any prejudice against the LGTB community during his term.^17^ If, on the one hand, he maintained in his campaign a mild tone on the culture of the Carnival and the rights of the LGBT movement, on the other hand, he clearly maintained his position against the liberalization of drugs, the legalization of abortion, and the introduction of the gender ideology in schools, associating the defense of family values with a natural construction of citizenship.

Already during his term as mayor, Marcelo Crivella started mingling the religious and the political to such an extent that it made him accumulate some processes for administrative improbity due to making use of the administrative machine for the benefit of the Pentecostal churches and faithful, for instance by making some public services more available to them than to other social groups and helping his own church enter the municipal schools and local communities with its proselytizing social assistance programs.^18^ His impeachment process opened in July 2018^19^ as a result of a “secret meeting” with a group of (neo)Pentecostal pastors and pre-candidates to the Congress, to whom Crivella indicated an easy access to medical aid, such as cataract and varicose veins surgeries, as a courtesy from the municipal government to Pentecostal communities and prospective voters.^20^ The Public Ministry also investigates the control of Afro-Brazilian cults’ events with a veto power directly by the office of the mayor. Gomes and Leite (2019: 87) analyzed the decree 43.219/2017 and its 50% cut in the public funds for the Carnival^21^ while requiring a permit to organize the samba and Afro-Brazilian religious activities in public places. The cut in the budget was justified by the fact that it would be transferred to municipal daycare centers. On the one hand, it generated accusations of religious intolerance and the demonization of popular culture, but on the other, such “a political calculation of risk” generated benefits with electoral repercussions (Vital da Cunha 2017), winning the sympathy of many people, especially in the IURD target market, in need of such centers.^22^

Although the mayor received the title of World Heritage of UNESCO attributed to the Cais do Valongo^23^ in November 2018, throughout his mandate there were several vetoes to other projects that value places and rites constituting African cultural memory in Rio de Janeiro, for instance, to transformation of the quilombo of the salt stone into immaterial heritage,^24^ to renovation of the iabás fair,^25^ to the funds designated for the jongo da serrinha^26^ and the feasts of Iemanjá, and an introduction of excessive bureaucratization to organize these events in the public space. Such problems have generated reactions in defense of the important Afro-Brazilian elements constituting intangible cultural heritage of the city (Gomes and Leite 2019).

## Rio de Janeiro Under Crivella: Political Bargaining in the Shadow of the Pandemic Crisis

The challenges faced by all political leaders in the time of the COVID-19 pandemic are multiple. On the one hand, it is a great responsibility to help protect those who are vulnerable, likely to suffer serious health implications due to the impact of the virus, which has been scientifically proven to be community-spread; on the other hand—virus is not lethal to the vast majority of population, and global statistics show that most of the infected get over it, sometimes even asymptomatically. There is also another social and economic background to the pandemic crisis: lockdown and quarantine policies promoting social distancing can, in fact, be effective in lowering the curve of infections and thus mortality rate of those who succumb to this illness. But quarantine and implementing the “stay at home” policy can really happen only when people actually live in conditions allowing them for social distancing, which is not the case of the majority of Rio de Janeiro inhabitants. Additionally, focusing all public health policies on the virus increases signs of collapse in the areas of health care other than epidemic, lowering their capacities, especially for urgent life-saving and health saving operations. Last but not least—in the lockdown situation, there is an urging necessity to provide for those who have been left with no jobs, no adequate health insurance, and no means to live on and whose choice is between suffering from virus or starvation. Too many in Rio de Janeiro still survive on too little, which the pandemic has made even less. Therefore, both the virus expansion and the situation of the lockdown and quarantine have nowadays been tough on many, provoking public disputes and many controversies, involving also the decisions of the current mayor of the city of Rio de Janeiro. In accordance with the data of IBGE^27^ (and the compensational theory of existential security), the target group consists mostly of the weakest in the structure of Rio de Janeiro population—when it comes to the statistics, people describing themselves as Pentecostals (Evangelicals) have rather “dark” skin (45.7%), are not well educated (42.3% of those over 15 years of age have not completed their primary education and 8.6% are illiterate), and earn little money (as many as 63.7% earn below one minimum salary). The majority of them are females, many employed informally, especially in domestic services sector, paid per day for the service done. Many are not possessing health insurance or any social security. Therefore, they are precisely the ones who, in the pandemic situation, have no means to protect themselves and their families and no prospects for going online with their manual jobs. All in all, those for whom the lockdown situation in the city means problems and their problems mean decrease in the IURD (and other Pentecostal sector churches) resources—the income of the church is based mainly on its followers’ contributions (the obligatory tithe and other, voluntary donations). In order to protect the stability of their institution, its leaders must convince the followers to be loyal and generous enough, even in the times of pandemic—for this reason, according to the market theory of religion, the rewards and compensations they offer must be attractive and the church must show its support and understanding for their situation.^28^ This must lead to the necessity of adjustment of not only teachings, but also public policies to meet the followers needs and expectations. It is one of the reasons, together with national and local political alliances, for the city of Rio de Janeiro experiencing, between March and June 2020, a very complex political and religious intertwining, with the dialectic relations between the federal government (President Bolsonaro), state government (Wilson Witzel), and Rio de Janeiro city hall (Marcelo Crivella) marking the time in the face of political and religious demands on COVID-19.

The Federal Supreme Court (STF) guaranteed, against the intentions of the federal government of president Bolsonaro, autonomy for each state to organize itself against the pandemic,^29^ the decision which has created situations of the local uncontrollability of the pandemic due to the lack of the centralization of the COVID-19 combat policies at the national level, which, in many cases, including Rio de Janeiro, led to growing pressures on the state and city government exercised by their political allies, local business, militiamen,^30^ and religious institutions, provoking discussions and controversies on the necessity of implementing the rules of social isolation, the necessity of closing the commerce, and other social and economic restrictions. If, on the one hand, at the first moment, the state government of Rio, under Wilson Witzel’s command, was against giving preference to the economy instead of the quarantine, on the other hand, Mayor Crivella continued supporting President Bolsonaro as his church ally and loosening the rules of the quarantine imposed by the state government, which officially instituted lockdown. In practice, Crivella made it flexible, according to president Bolsonaro’s liberal “Swedish style”^31^ policies, which resulted in the lenient control of the public places, and instead opting for distributing paper masks^32^ and maintaining open various local businesses spread throughout the city, including violent Baixada Fluminense, controlled by local militia gangs.^33^

The city, in spite of the fact that it was officially quarantined and locked down in accordance with the state law, started suffering from the increasing infection curve. In such a situation, however, both the governor (accused, in the meantime, of corruption in managing the construction of COVID-19 hospitals)^34^ and the mayor (supporting the federal government in defense of the vertical social isolation and the necessity of opening the local economy to prevent unemployment and poverty among the most vulnerable part of the city population and, at the same, the biggest target market of the IURD)^35^ soon “aligned” themselves in the policy of relaxation of the official lockdown, to comply with the daily economic needs of the majority of the city population.

The conflict between the state and the city hall in Rio de Janeiro, most visible during the initial period of the pandemic, marked by an attempt of the lockdown implementation, might have been a reflection of the federal level competition between the two incumbents: both president Bolsonaro, supported by Crivella and his church, and the state governor Witzel are mentioned as candidates to (re)election in the next presidential contest. In order to show his support for Bolsonaro, Edir Macedo (and Silas Malafaia from another Pentecostal denomination—Assembleia de Deus Vitória em Cristo) initially rejected the suspension of religious cults and continued organizing religious meetings, viewing the virus as devils’ and media invention directed at promoting social panic and showing disbelief in the gravity of the pandemic situation. At the same time, Evangelical congressmen insisted on opening the temples, giving theological arguments for the necessity of mass prayers in order to face the pandemic, and, eventually, proposing ways of controlling the number of people participating in the cults. However, soon their attitude was changed or attenuated due to the decisions of many other (neo)Pentecostal pastors declaring the necessity of maintaining social distancing (churches such as: Cristã Nova Vida, Renascer em Cristo, Batista da Lagoinha, Sara a Nossa Terra, Cristã Maranata, Evangelho Quadrangular, Assembleia de Deus Betesda), which was an attitude shared by other sectors of the religious market: Catholics, Presbyterians, Baptists, Methodists, and Lutherans. They all suspended their communitarian service activities, offering online substitutes instead and persuading their faithful to follow the social distancing “stay at home” rules.^36^ This situation made both Macedo and Malafaia retreat from their initial standing and offer social assistance to those in need of help in the times of pandemic.

Soon, however, Edir Macedo was admitted to the Moriah hospital with COVID-19 symptoms, where he stayed for 4 days receiving successful treatment with Chloroquine^37^—controversial medicine, strongly suggested by the federal government and many times mentioned in public appearances by president Bolsonaro himself. On his recovery, Macedo confirmed the positive effects of the medicine, in accordance with his ally’s opinion, and reasserted that strong faith is essential for combating the virus.^38^

In the meantime, the necessity of quarantine was being questioned by local courts, and there was a lot of controversy among the representatives of scientific authorities of the Rio de Janeiro State. Due to political bargaining (for and against the steps of the crisis management suggested by the federal government) and failing state management, there happened many personal changes at the state level governed by Witzel, including some due to the accusations of irregularities in pandemic hospital construction, as well as dismantling a crisis management team with participation of doctors and researchers who were supposed to advice on the matters of the pandemic.^39^

In the chaotic situation, mayor Crivella and his city hall started investing in social aid, especially directed at the poorest inhabitants of the city, for instance making donation of tomography and other medical equipment for the poorest boroughs of the city, including the inhabitants of the biggest favela Rocinha and, in this particular case, installing it in the interiors of the IURD temple, which provoked an immediate reaction of the political opponents from the leftist PSOL demanding explanations.^40^ Crivella defended his decision by means of presenting it as a temporary solution, which enabled quick diagnostics to save lives and promised that the equipment would end up at the local hospital after the pandemic. At the same time, just as mayors of other big cities, he was faced with the problem of the shortage/lack of tests for the virus in the city population^41^—the rate of testing was low, and the absence of tests resulted for some time in statistics that did not confirm the gravity of the COVID-19 pandemic, as most deaths were registered as “acute respiratory syndrome” or “flu.” Sometimes statistics were not available at all or the methodology of counting suddenly changed or were substantially delayed, which has made it difficult to follow the data and analyze them in order to make an objective evaluation of the scope of the pandemic and the activity of the mayor in relation to it.^42^

One of the major problems that researchers face is precisely to access reliable and current data and distinguish among various sources of information that is available on Internet, while analyzing it in a scholarly way, with no prejudice towards any political option (especially in view of the ongoing media war), but at the same time with full consideration for the importance of the religious factor salient in local public space. The amount of fake but apparently “scientific” news is overwhelming and makes it difficult to find the true picture of pandemic processes and events. Half-truths, facts, and words taken out of the context make it difficult to see what is a purely religious argumentation, what is purely political, and what is purely scientific.

The pandemic management is a controversial and unnecessarily politicized issue: to some, the prospective “opening of the city,” justified by Crivella by means of the apparent successful control over the situation, is just an example of wishful thinking and a decision bearing too much risk for the urban population; to others, a self-fulfilling prophecy, just like in Merton’s (1970) classical analysis; and to many others (especially Crivella’s political and religious followers and prospective voters posting acknowledgements on his facebook account),^43^ a deserved end of the pandemic crisis, well-managed by the city hall in accordance with the federal level–suggested strategies. What needs to be stressed, however, is that there has been no consensus even within the Pentecostal community on acceptable/desirable ways of dealing with the pandemic crisis and the attitudes that should be taken and supported.^44^

## Conclusions

As we have shown, for more than 30 years already, the Neo-Pentecostal IURD has been an important part of the public sphere in Brazil, present in various elections, increasing its partnerships (or competition) with the State, in compliance with alliances built by Edir Macedo, which puts its leaders in a clear contrast to the Luckmannian process of the increasing “invisibility” of religion. As we showed in our paper, also Crivella’s policies as the mayor of Rio de Janeiro mingle the religious and the political in order to strengthen his church, currently aligning his policies with the federal government, even against the Rio de Janeiro state governor. The IURD’s leaders have chosen, not for the first time, a strategy of acting in close alliance with the federal government, supporting it politically and, at the same time, implementing their own institutional growth policies, which influence, to a large extent, the bishop/mayor’s decisions and management tactics in the city of Rio de Janeiro, including the times of the pandemic, which, just like other periods of social crises, exacerbate the importance of political leadership linked to the “field of religious production,” determined by a set of dispositions, patterns of thinking, and ways of perceiving the world characteristic of a given segment of society. As there are many different ways of approaching the concept of “leadership” in academic research, we chose to see it in a systemic context, concentrating mostly on its background and practical functioning. That is why we showed how Marcelo Crivella—a bishop of IURD and a nephew of Edir Macedo, was supported from the beginning of his political career, first in congressional elections, then in the ministerial duties at the federal government level, and finally in his successful Rio de Janeiro mayor’s elections by his church.

Notwithstanding the already noticeable presence of the IURD at all levels of political representation in Brazil, Crivella’s prominent position in the “Marvelous City” has been, undoubtedly, a major victory and excellent publicity for his church, due to the global importance of the city he is governing. Therefore, his performance has been closely watched not only by his own church leaders and affiliates. As one of the most important functions of any leader is the ability to lead his/her supporters through and out of the stormy periods of crisis (here, pandemic) requiring sometimes the use of emergency measures (Weber 1946, 2002), any attempt to assess Crivella’s performance, though difficult before the end of his term, not only seems to be an interesting task from a purely academic point of view but also constitutes an important predictive factor in terms of a possible personal (Crivella’s) and institutional (IURD) future political involvement at the higher governmental levels. Bishop/Mayor Crivella and his Rio de Janeiro management can be looked upon as an important part of the strategies of growth implemented by the IURD charismatic leader Edir Macedo. The IURD has been for decades already among the fastest growing religious institutions in Brazil, promoting its image and recognition via own TV stations, publishing houses, and politicians—its high-rank members and various political allies have been supported institutionally in elections, disseminating the perception of the IURD as an important piece of the Brazilian political landscape. Leadership-strong, result-oriented, centralized church, with a vertical hierarchy, efficient from the managerial point of view and with disciplined followers-voters, is indeed a political ally in high demand, implementing new rules of conduct in Brazilian politics. One of them is the rule of “brother votes for brother,” which means disciplined supporting the representatives and allies in electoral campaign, participating in the political struggle and conflicts arising from it.

Rational attitudes often find themselves at a loss when confronted with the reality of life, habits, and human conviction or a direct impact of political decision on the personal situation. All over the world, political leaders have been faced with similar dilemmas, challenges, and prospects due to the COVID-19 epidemic: a need for saving and protecting lives; implementing health system emergency; and reacting to the economic downturn, unemployment growth, and collapse of many family households due to the lockdown policies, which creates difficult challenges for the labor market, if implemented. The pandemic hurts especially unequal societies like Brazil, proving double standards of local quarantine policies, which have become controversial: it was visible in Rio de Janeiro during the lockdown when strict regulations, on the one hand, included legal restrictions on contacts between people and, on the other hand, could not help numerous families staying, as always, in a single-room space. Orders, prohibitions, and sanctions linked to the quarantine do not work in the context of poverty, decrepit housing conditions, limitations of access of low-income population to hospital treatment, adequate diagnosis, lack of tests, and poorly recognized state of the intensity of the epidemic in densely populated favelas, where people are particularly vulnerable to the transmission of the disease. In the city of Rio de Janeiro, there are also many difficulties in transferring the methods of fighting a pandemic between various areas of the city, mainly due to different social and economic status of their inhabitants and, consequently, their living conditions.

Choosing between maintaining the requirements of isolation leading to the interruption of the transmission of the disease and the formation of an attitude of a vertical isolation or “herd resistance” towards it is a difficult challenge for any leader, just like managing the increase in stress resulting from the prolongation of tensions, uncertainty, fatigue, and anxiety of the masses of Rio de Janeiro inhabitants, particularly true for those who are at risk of domestic violence. All these problems have put the city of Rio de Janeiro in an emergency situation in the pandemic context, with religion, politics, and science disputing their own truths and solutions. The pressures on local government from social, religious, and economic groups show clearly how these segments either cooperate or fiercely compete against each other, sometimes denying scientific advances, turning facts into opinions and vice versa, and manipulating public opinion through friendly or own media. Such phenomena have become visible in Crivella’s Rio, for instance in relation to the controversies about social isolation rules, access to health on the part of the poorest or medicine used in COVID-19 treatment (Chloroquine), etc. The situation of pandemic, just like any other crisis, shows with clarity both the problems of the scientific and the secular clashing with the religious and the emotional or, quite on the contrary, the two spheres mixing and mingling till they become almost inseparable.

## Footnotes

1 The nature of the notion of leadership can be outlined by indicating at least three main approaches to this phenomenon: attributive, relational and functional. The attributive approach assumes that leadership is a set of specific properties of an individual, combined with a unique gift (charisma), which inspires admiration, enthusiasm, respect and devotion of the followers. The relational approach focuses on leadership as a process of relationship building dependent on psychosocial mechanisms activating values, beliefs and actions by means of authority, personal charisma, but also manipulation, depending on cultural and institutional patterns in the group, as well as individual characteristics of leaders. The third approach is functional, based on the perception of institutional leadership building the identity, defining the mission, setting strategic goals, as well as personal commitment. This approach is not directly linked to a particular person, but more to a given institution. The above approaches are not mutually exclusive.

2 See current data on the COVID-19 in Brazil: https://www.bbc.com/portuguese/internacional-51718755

3 The data are from the state government of Rio de Janeiro. See: http://painel.saude.rj.gov.br/monitoramento/covid19.html?fbclid=IwAR3emWQZx6AEooqNUGT6lTt_xXCkOqPU3tlaxf-a01BpVCypo60zULf0jEY

4 https://countryeconomy.com/gdp/brazil

5 https://www.indexmundi.com/facts/brazil/indicator/SI.POV.GINI

6 The IURD is one of the most powerful Brazilian Neo-Pentecostal from the financial standing point of view. The church’s new Cathedral in São Paulo (The Salomon’s Temple) belongs to the biggest temples on the continent and the founder’s fortune amounts to millions of USD (Antunes 2013).

7 Such conflicts often end up with dissents—the first one in the history of the IURD was with Romualdo Suares, Macedo’s brother-in-law, who decided to start his own church (Internacional de Graça de Deus); the latest one, in came with the departure of pastor Romualdo Panceiro, for a long-time number two of the church, who also decided to found his own church. Before him, pastor Valdemiro Santiago, had also successfully founded his own church—Mundial do Poder de Deus. See: https://www.terra.com.br/noticias/brasil/de-sucessor-oficial-de-edir-macedo-ex-numero2daigrejauniversalviracompetitor,01ceac0fde2b23638e9df0abcb464390z6gqdu7d.html?fbclid=IwAR0E_aUmelmxQ1QRNRFD8rzU06C4-YpmS9uxr9cz29rNIR9rejnSjJQDESE.

8 The Republican Party has recently acquired new members—two sons of the president Bolsonaro (Flávio e Carlos Bolsonaro, who are Baptists). The party has currently 33 federal congressmen (out of 512), 2 senators (out of 81), 42 state congressmen (out of 1.024), 106 mayors (out of 5.570), 1606 city councellors (out of 56.810). https://www.camara.leg.br/; https://www25.senado.leg.br/web/senadores/em-exercicio/-/e/por-partido

9 The church has got its “*daughter companies”* almost all around the world. Marcelo Crivella, at the beginning of his term as mayor, traveled to Africa in April 2017 and participated in an Easter service of the Universal Church at Ellis Park Stadium in Johannesburg, later preaching in Durban and Cape Town. The video of his appearance was posted by his wife, Sylvia Jane Crivella, on his Facebook profile. Crivella was announced as a bishop and greeted by a crowd of people. Next to him, Bishop Marcelo Pires appeared. The event was attended by the president of South Africa, Jacob Zuma. Accessed: 16 Jun 2020: https://oglobo.globo.com/rio/marcelo-crivella-comenta-pascoa-em-culto-na-africa-viagem-pessoal-os-gastos-tambem-21212852 and https://blogs.oglobo.globo.com/ancelmo/post/crivella-pregara-e-cantara-em-zulu-em-tres-estadios-da-africa-do-sul-nesta-pascoa.html.

10 See: https://www.facebook.com/linerecordsbrasil/. Accessed: 20 Jun 2020. About the importance of gospel music for the IURD strategies of growth, see, for instance, Rosas (2013).

11 Garotinho (mayor of the city of Rio de Janeiro from 1999 to 2002) converted to Pentecostalism in 1995, currently belongs to the Presbyterian Church of Brazil (IPB). In 2002, he made a Pentecostal tour of Brazil to build support for his own presidential elections. During his term as the mayor of Rio de Janeiro he passed a state law nr. 3.459, which determined the implementation of religious education at public schools, provoking controversies. Access: 20 Jun 2020: https://www1.folha.uol.com.br/fsp/brasil/fc29039815.htm; https://www1.folha.uol.com.br/fsp/brasil/fc0711199912.htm; http://www.conectabaixada.com.br/politica/2016/11/18/pastor-revela-segredos-de-garotinho/; https://gov-rj.jusbrasil.com.br/legislacao/136999/lei-3459-00. Acting as Crivella’s political coach, Garotinho points at the growing professionalism of the IURD due to implemention of internal “ecclesiastical and theological” reforms, which made it much more sophisticated, “more liberal and pragmatic, comparable to religious conservatives of the Republican Party in the United States*,* more appealing to the middle class, business and opinion forming circles”. Accessed: 16 June 2020: https://www.bbc.com/portuguese/brasil-37829549.

12 http://www.projetonovacanaa.com.br/nossa-historia/. Accessed: 20 Jun 2020.

13 PSOL (the Socialism and Freedom Party) is a Brazilian left-wing political party founded in June 2004. Its creation was driven by dissidents from the Workers’ Party who claimed to disagree with the party’s policies.

14 For more elaborate analysis on the reasons for Freixo’s electoral defeat, see interview with Ricardo Mariano. Access: 16 June 2020: https://brasil.elpais.com/brasil/2016/10/31/politica/1477940246_927730.html

15 Access: 16 June, 2020: https://oglobo.globo.com/brasil/slogan-de-crivella-surgiu-como-contraponto-gestao-de-paes-20393473

16 See book: Plan of Power: God, Christians and Politics, by Bishop Macedo, published in 2011, in it there is a clear project of theocracy.

17 Accessed: 16 Jun 2020: https://www.bbc.com/portuguese/brasil-37795802

18 Accessed: 15 Jun 2020: https://noticias.uol.com.br/politica/ultimas-noticias/2018/07/12/mp-acusa-crivella-de-usar-escolas-publicas-para-eventos-da-universal.htm

19 Accessed: 18 Jun 2020: https://oglobo.globo.com/rio/2018/07/10/2272-crivella-faz-articulacoes-politicas-para-evitar-processo-de-impeachment

20 Accessed: 15 Jun 2020: https://g1.globo.com/rj/rio-de-janeiro/noticia/ministerio-publico-do-rio- com-acao-na-justica-contra-o-prefeito-marcelo-crivella.ghtml

21 Accessed: 24 Jun 2020: http://g1.globo.com/rio-de-janeiro/carnaval/2018/noticia/escolas-de-sambacontinuam-em-busca-de-solucoes-para-viabilizar-o-carnaval-de-2018.ghtml; https://oglobo.globo.com/rio/envolvido-em-polemicas-em-torno-da-foliacrivella-alvo-de-criticas-nos-sambas-dos-blocos-de-carnaval-22347754.

22 See: Christina Vital in *Heinrich Böll Stiftung*. 01/11/2017. Accessed: 24 Jun 2020: https://br.boell.org/pt-br/2017/11/01/ha-calculo-politico-nas-afirmacoes-de-crivellaafirma-christina-vital-em-entrevista.

23 https://diariodoporto.com.br/cais-do-valongo-um-patrimonio-mundial-sob-risco/ http://cultura.gov.br/unesco-entrega-titulo-de-patrimonio-mundial-para-o-cais-do-valongo-2/

24 The Pedra do Sal Quilombo is located in the port area of the city of Rio de Janeiro, around Pedra do Sal, João da Baiana Square and São Francisco da Prainha Street. With a history of slave resistance, the quilombolas claim territory called Little Africa, traditionally occupied since the time of their ancestors, which became the cradle of samba and the first samba schools of carnival.

25 A meeting of samba and typical cuisine from the suburbs of Rio de Janeiro, the Yabás Fair gathers tents of 16 aunts from Madureira who prepare delicious recipes from Afro-Brazilian gastronomy. Yabá, which means Mother Queen, is the term used in Afro Brazilian religions to define all female orishás.

26 With the end of slavery in the nineteenth century, many former slaves migrated from the farms in the Paraíba River Valley to Rio de Janeiro, transforming the city into one of the regions of the country with the highest concentration of jongueiros on the hills of São Carlos, Mangueira, Serrinha and Salgueiro.Jongo da Serrinha, in Madureira, is the most traditional drumming group in Rio, created in the late 60s by Master Darcy and Grandma Maria.

27 Data from the last National Survey of the IBGE from 2010: www.ibge.gov.br

28 Due to the necessity of closing the temples, the financial situation of many (neo)Pentecostal churches and their pastors has deteriorated (including IURD), which made them ask for the president’s Bolsonaro intervention. See: https://economia.uol.com.br/noticias/estadao-conteudo/2020/04/30/bolsonaro-pressiona-receita-federal-a-perdoar-dividas-de-igreja-evangelica.htm; https://domtotal.com/noticia/1414543/2020/01/para-agradar-evangelicos-bolsonaro-quer-pagar-conta-de-luz-de-igrejas-com-dinheiro-publico/; https://brasil.elpais.com/brasil/2019-12-26/igrejas-devem-mais-de-460-milhoes-de-reais-ao-governo.html; https://www.cartacapital.com.br/blogs/dialogos-da-fe/quando-lideres-religiosos-barganham-no-mercado-politico/; https://piaui.folha.uol.com.br/sem-fieis-sem-dizimo-sem-palanque/ e https://economia.uol.com.br/noticias/estadao-conteudo/2020/04/30/receita-aplica-pesadas-multas-a-igrejas.htm Accessed:13 Jun, 2020

29 Accessed: 17 Jun 2020: https://www12.senado.leg.br/noticias/materias/2020/04/16/decisao-do-stf-sobreisolamento-de-estados-e-municipios-repercute-no-senado

30 Militiamen are armed criminal organizations, consisting mostly of ex-policemen, ex-soldiers and prison guards acting in favelas, selling security and ilegal services such as clandestine electricity connections, etc. The militia business is often supported by politicians and community leaders, due to their political and business connections (Alves 2003). Accessed: 23 Jun 2020: https://exame.com/brasil/alves-da-ufrj-milicia-tem-poder-maior-que-o-trafico-no-rj/

31 The Swedish government opted for not introducing officially any quarantine and restrictions and instead betting on the “herd immunity” acquisition of the population after the exposure to the virus.

32 Accessed: 24 Jun 2020: https://www1.folha.uol.com.br/cotidiano/2020/05/prefeitura-do-rio-distribui-mascara-de-papelao-contra-virus-e-e-criticada.shtml

33 Militiamen supported the opening of local businesses, which made it possible for the gangs to keep their income from decreasing. Access: 20.06.2020:https://www.brasildefato.com.br/2020/04/22/pesquisa-relaciona-aumento-de-covid-19-com-atuacao-de-milicias-na-baixada-fluminense; https://dmjracial.files.wordpress.com/2020/04/boletim-vii-2020.pdf; https://noticias.uol.com.br/cotidiano/ultimas-noticias/2020/04/17/policia-investiga-reabertura-de-comercio-a-mando-de-milicia-no-rio.htm/https://rioonwatch.org.br/?p=47487

34 Accessed: 20.06. 2020: https://www.correiobraziliense.com.br/app/noticia/politica/2020/05/29/interna_politica,859372/investigado-por-corrupcao-na-saude-witzel-pede-ao-stj-para-depor-a-pf.shtml**.**

35 Accessed: 24 Jun 2020: https://noticias.uol.com.br/saude/ultimas-noticias/redacao/2020/06/08/flexibilizacao-de-witzel-tem-baixa-adesao-na-regiao-metropolitana-do-rj.htm; https://noticias.uol.com.br/ultimas-noticias/agencia-estado/2020/06/08/justica-do-rio-suspende-flexibilizacao-autorizada-pela-prefeitura-e-estado.htm; https://oglobo.globo.com/rio/taxa-de-contagio-da-covid-19-no-rio-volta-subir-preocupa-especialistas-24495435; https://www.poder360.com.br/coronavirus/fernando-ferry-pede-demissao-da-secretaria-de-saude-do-rio/.

36 See: https://www.cartacapital.com.br/blogs/dialogos-da-fe/diante-da-crise-do-coronavirus-o-que-as-igrejas-podem-fazer/ Accessed: 15 Jun 2020.

37 See:https://jovempan.com.br/noticias/brasil/internado-covid-19-edir-macedo-tratamento-cloroquina-recebe-alta.html Accessed 15 Jun 2020

38 Sócrates Oliveira de Souza, the head of Convenção Batista Brasileira (the first lady, Michelle Bolsonaro is a member of the church Atitude belonging to the association), also was cured by means of the same medicine and publicly confirmed its positive impact on his recovery. About Macedo’s treatment, see: https://veja.abril.com.br/brasil/universal-comemora-cura-da-covid-de-edir-macedo-por-hidroxocloroquina/ e https://mblnews.org/nacional/bispo-edir-macedo-e-diagnosticado-com-covid-19?fbclid=IwAR0UImGtQHqHCjtzf-TVKQqhCLYzkEJ45nk1QSR_BT_VeZeU_Or5wrBBbvU Accessed: 15 Jun 2020

39 https://noticias.r7.com/rio-de-janeiro/apos-crises-no-governo-do-rj-witzel-faz-mudancas-no-secretariado-30062020, accessed: 30 Jun 2020

40 See:https://g1.globo.com/rj/rio-de-janeiro/noticia/2020/05/06/mp-analisa-decisao-de-crivella-de-instalar-tomografo-em-igreja-universal-medida-foi-criticada-por-moradores-da-rocinha.ghtml Accessed: 14 Jun 2020

41 However, we must also point out, that the lack of access to tests has been a common problem in many parts of the world and, due to the increased demand and very low supplies, prevention or diagnostic testing has been introduced in very few countries.

42 See: https://noticias.uol.com.br/saude/ultimas-noticias/redacao/2020/03/23/casos-suspeitos-registrados-como-gripe-em-favela-do-rio-denunciam-medicos.htm. Accessed: 14 Jun 2020. See:https://oglobo.globo.com/rio/prefeitura-do-rio-tira-do-ar-dados-abertos-sobre-mortos-pela-covid-19-no-municipio-24434182 e https://noticias.uol.com.br/saude/ultimas-noticias/redacao/2020/05/28/rio-1177-mortes-somem-de-estatistica-de-covid-e-especialistas-criticam.htm; https://g1.globo.com/rj/rio-de-janeiro/noticia/2020/05/09/rj-tem-quase-20-vezes-mais-internacoes-por-sindrome-respiratoria-aguda-do-que-em-2019-diz-estudo.ghtml. Accessed 14 Jun 2020; https://noticias.uol.com.br/saude/ultimas-noticias/redacao/2020/05/27/rio-metodologia-mortes-coronavirus.htm ou https://piaui.folha.uol.com.br/lupa/2020/05/14/covid-19-dados-registro-civil-sobre-mortes/ Accessed 14 Jun 2020

43 https://www.facebook.com/marcelocrivella/, accessed: 18 Jun 2020

44 See https://www.bbc.com/portuguese/brasil-52313890 . Accessed 14 Jun 2020

## References

[CR1] Alves CE (1998) Lula agora elogia Ulysses e Edir Macedo, In: *Folha de São Paulo.* http://www1.folha.uol.com.br/fsp/brasil/fc01029812.htm. Accessed: 20 Jun 2020

[CR2] Alves JCS (2003). Dos barões ao extermínio: uma história da violência na Baixada Fluminense.

[CR3] Antunes A (2013) The richest pastors In Brazil, *Forbes*. https://www.forbes.com/sites/andersonantunes/2013/01/17/the-richest-pastors-in-brazil/#7-cec3b065b1e. Accessed: 20 Jun 2020

[CR4] Bourdieu P (1991). Genesis and structure of the religious field. Comparative Social Research.

[CR5] Burity J (2018) A onda conservadora na política brasileira traz o fundamentalismo ao poder? In: Almeida R; Toniol R (orgs.) *Conservadorismos, fascismos e fundamentalismos: análises conjunturais*. Editora da Unicamp, Campinas

[CR6] Burns JM (1978). Leadership.

[CR7] Camurça MA (2020). Igreja Universal do reino de deus: entre o “plano de poder” e a lógica de minoria perseguida. Religião e Sociedade, Rio de Janeiro.

[CR8] Castro D (2017) Genro do Edir assume Universal e manda ex-chefão da Record pregar na África, In: *ntv. Notícias da TV.* Retrieved from: https://noticiasdatv.uol.com.br/noticia/televisao/genro-deedir-assume-universal-e-manda-ex-chefao-da-record-pregar-na-africa%2D%2D17186?cpid=txt. Accessed: 20 Jun 2020

[CR9] Ciulla JB (2004). Ethics, the heart of leadership.

[CR10] Edwards GC (2000) Does the messenger matter? The role of charisma in public leadership, In: Congress and Presidency*,* vol.29, nr 1, pp. 25–46

[CR11] Fernandes RC (1998). Novo nascimento: os evangélicos em casa, na igreja e na política.

[CR12] Ferrari OA (2007). Bispo S/A.

[CR13] Freston P (2006). *Religião e política, sim; Igreja e Estado, não:* os evangélicos e a participação política.

[CR14] Goleman D (1998). What makes a leader*?*. Harv Bus Rev.

[CR15] Goleman D (2000). Leadership that gets results. Harv Bus Rev.

[CR16] Gomes EC, Leite MST (2019)A religião no poder executivo: controvérsias sobre “cultura” no mandato de Crivella no Rio de Janeiro In Religare,v.16, n.1, p.85–116

[CR17] Greenleaf RK (1977). Servant leadership: a journey into the nature of legitimate power and greatness.

[CR18] Machado MDC (2006). *Política e religião:* a participação dos evangélicos nas eleições.

[CR19] Mariano R (1999). Neopentecostais: sociologia do novo pentecostalismo no Brasil.

[CR20] Mariano R, Oliveira R (2009). O senador e o bispo: Marcelo Crivella e seu dilema shakespeariano. *Interações: Cultura e Comunidade (Faculdade Católica de Uberlândia),* Uberlândia.

[CR21] Mariano R, Pierucci AF (1992). O envolvimento dos pentecostais na eleição de Collor. São Paulo, Novos estudos Cebrap número.

[CR22] Merton R (1970). Sociologia: teoria e estrutura.

[CR23] Myers DG (2007). Exploring social psychology.

[CR24] Norris P, Inglehart R (2006). Sacrum i profanum. Religia i polityka na świecie.

[CR25] Oro AP (2005-2006) Neopentecostalismo macumbeiro. In: REVISTA USP, São Paulo, n.68, pp. 319–332, dezembro/fevereiro 2005–2006. Disponível em: http://www.revistas.usp.br/revusp/article/view/13505/15323

[CR26] Rosas N (2013). Religião, mídia e produção fonográfica: o Diante do Trono e as disputas com a Igreja Universal, *Religião e Sociedade*. Relig soc.

[CR27] Rost J (1991). Leadership for the twenty first century.

[CR28] Santos Ch, Da Escóssia F (2002) Bispos da Universal decidem apoiar Lula, In: *Folha de São Paulo,* http://www1.folha.uol.com.br/fsp/brasil/fc0810200241.htm. Accessed: 20 Jun 2020

[CR29] Siuda-Ambroziak R (2017) Religious and political leadership in Brazil at the turn of the 19th and 20th centuries – the case of Father Cícero”, Revista Brasileira de História das Religiões (dossiêr: As crenças e suas articulações com a política e a sociedade) IX/21(9), 9–20; http://www.periodicos.uem.br/ojs/index.php/RbhrAnpuh/article/view/34209/18107

[CR30] Siuda-Ambroziak R (2019) Prosperity gospel on the rise - development and expansion of neo-Pentecostal churches in Brazil, Ramos de Andrade, S, Siuda-Ambroziak, R, Stachowska, E (ed), Brazil-Poland. Focus on Religion, UEM, Maringá/UW, Varsóvia, 315–340

[CR31] Siuda-Ambroziak R, Stachowska E (2017) Religious market and its entrepreneurs: comparative perspective on Brazil and Poland. pp 2, 24–44 (**70**)

[CR32] Siuda-Ambroziak R, Stachowska E (2018) Relacje pomiędzy religią a polityką na przykładzie „kwestii aborcyjnej” – analiza porównawcza Polska-Brazylia In: Przegląd Religioznawczy*,* nr. 2(268)/2018, 171–194

[CR33] Stark R, Bainbridge WS (2007). Teoria religii.

[CR34] Stogdill RM (1974). Handbook of leadership: a survey of the literature.

[CR35] Vital da cunha C, Lopes PVL, Lui J (2017). Religião e política: medos sociais, extremismo religioso e as eleições de 2014.

[CR36] Weber M (1946) The sociology of charismatic authority, I. The general character of charisma. In: From Max Weber: Essays in sociology. Gerth H H, Wright C M (transl and ed) OUP, New York

[CR37] Weber M (2002). Gospodarka i społeczeństwo.

[CR38] Weis L (2008) Lula ‘deu a senha’ para o PT imitar Edir Macedo, In: *Observatório da Imprensa.* http://observatoriodaimprensa.com.br/codigo-aberto/lula-deu-asenha-para-o-pt-imitar-edir-macedo/. Accessed: 18 Jun 2020

